# Mechanical properties of the tumor microenvironment: drivers of immunotherapy resistance in solid tumors

**DOI:** 10.3389/fimmu.2026.1914878

**Published:** 2026-08-27

**Authors:** Zhen Zhang, Tianhao Deng, Wanshuang Zhou, Xiaoning Tan

**Affiliations:** Department of Oncology, The Affiliated Hospital of Hunan Academy of Chinese Medicine, Changsha, China

**Keywords:** cancer-immunity cycle, immunotherapy resistance, mechanical properties, mechanotransduction, tumor microenvironment

## Abstract

The limited efficacy of immunotherapy in solid tumors is increasingly recognized to reflect not only molecular and cellular immune suppression, but also profound mechanical abnormalities within the tumor microenvironment (TME). Emerging evidence indicates that extracellular matrix (ECM) stiffening and architectural remodeling, altered cellular stiffness, elevated solid stress, abnormal fluid shear stress, and increased interstitial fluid pressure (IFP) critically shape antitumor immunity and contribute to immunotherapy resistance. In this review, we summarize these major mechanical features and critically examine how they regulate the cancer-immunity cycle, including tumor-antigen release, antigen presentation, T-cell priming and activation, immune-cell trafficking and infiltration, tumor-cell recognition, and cytotoxic killing. We further highlight that tumor mechanical properties can function as mechanical immune checkpoints that promote immune evasion. Finally, we evaluate emerging strategies that target ECM remodeling, cancer-associated fibroblasts, vascular dysfunction, IFP, and mechanotransduction pathways to improve immunotherapy efficacy. The therapeutic evidence discussed is predominantly derived from preclinical studies. Overall, integrating tumor mechanical properties into cancer immunology provides a broader framework for understanding immunotherapy resistance and developing rational combination strategies for solid tumors.

## 1 Introduction

Solid tumors remain a leading cause of human mortality with incidence and mortality rates continuing to rise (1). Global estimates indicate that nearly 20 million new cancer cases and 9.7 million cancer-related deaths occurred in 2022 (2). Over the past few decades, advances in cancer immunotherapy, particularly the clinical application of immune checkpoint inhibitors (ICIs), have revolutionized the treatment landscape for solid tumors and produced durable responses in a subset of patients (3, 4). Nevertheless, primary and acquired resistance to immunotherapy remains a major clinical challenge, as many patients either fail to respond or eventually develop disease progression after an initial response (5).

**Figure 1:**
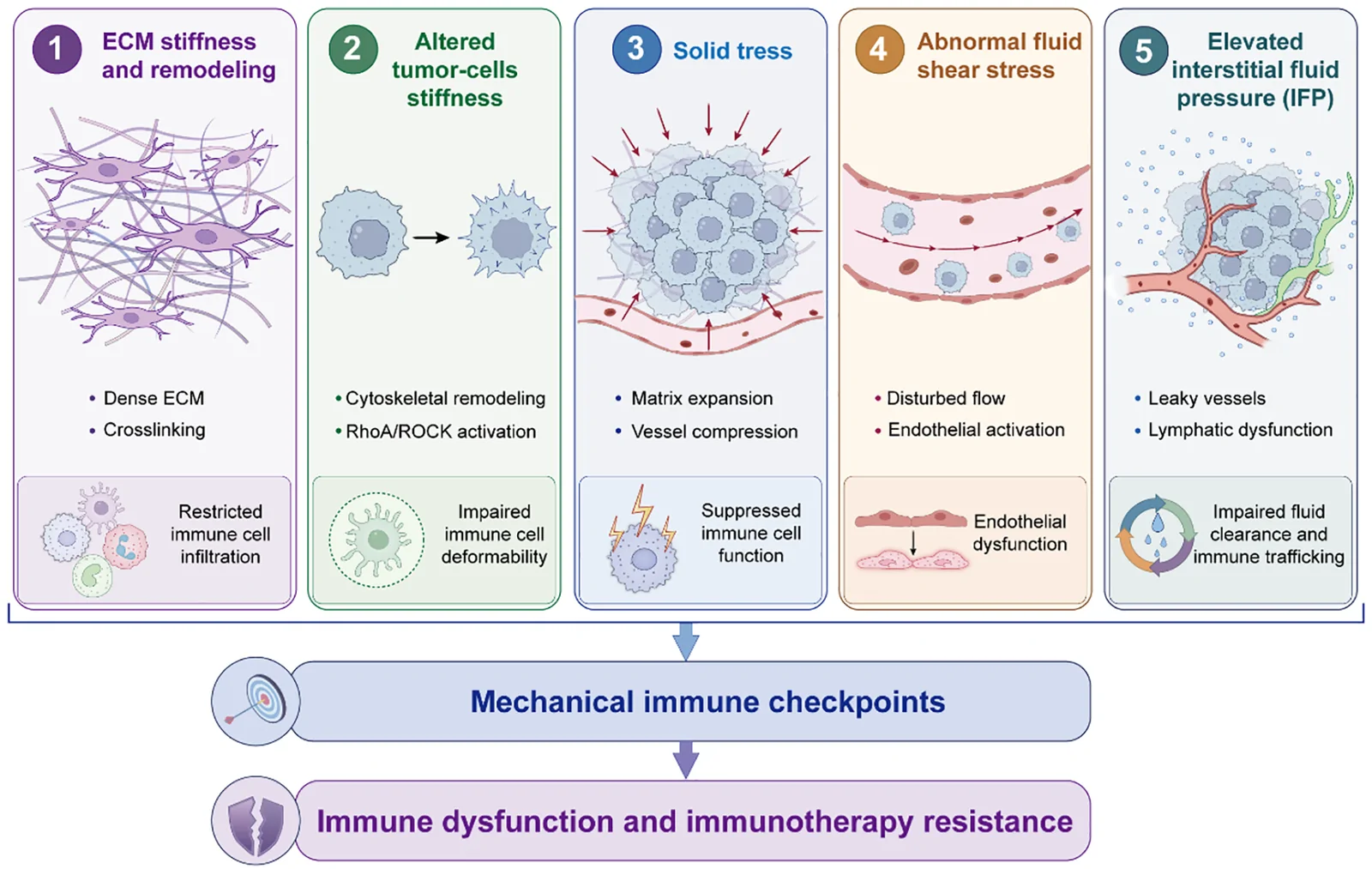
Mechanical abnormalities in the tumor microenvironment establish mechanical immune checkpoints and promote immunotherapy resistance. The figure was created using Adobe Illustrator.

Previous studies of cancer immunotherapy have largely focused on the dynamic interactions between tumor cells and immune components within the tumor microenvironment (TME) (6). However, these advances have not fully addressed the persistent clinical problems of limited efficacy and poor durability of response in solid tumors. In recent years, the mechanical properties of the TME have emerged as critical regulators of anticancer immunity and have therefore gained increasing attention in cancer immunotherapy (7). Accordingly, this review summarizes the major mechanical abnormalities of the TME, critically examines how these properties regulate the cancer-immunity cycle and contribute to immunotherapy resistance, and evaluates mechanically informed strategies for improving cancer immunotherapy. As most interventions targeting tumor mechanical properties remain at an early stage of development, the therapeutic discussion focuses predominantly on evidence from preclinical studies, while incorporating the limited translational and clinical evidence available. Therefore, tumor mechanics may represent an additional layer of immune regulation beyond molecular immune checkpoints (**Figure 1**).

## 2 Mechanical properties in the TME

Increasing evidence indicates that tumor development is accompanied by profound mechanical remodeling, including the progressive accumulation of extracellular matrix (ECM) (8), solid stress driven by cellular proliferation (9), and elevated interstitial fluid pressure (IFP) resulting from vascular dysfunction (10). These changes profoundly influence immune cell proliferation, immigration, infiltration and function (11, 12) (**Table 1**).

**Table 1:** Mechanical alterations within the tumor microenvironment shape antitumor immunity.

Mechanical feature	Main biological mechanisms	Effects on antitumor immunity	Representative evidence	References
Extracellular matrix (ECM) stiffness	Excessive collagen deposition, fiber alignment, HA accumulation, and LOX/LOXL2-mediated collagen crosslinking increase ECM stiffness.	Restricts immune cell migration, promotes T-cell exhaustion, and induces immunosuppressive signaling.	High collagen density reduces CD8+T cell infiltration and contributes to immunotherapy resistance.	(14, 18, 23, 30, 35)
Desmoplastic reactions (DRs) and cancer-associated fibroblasts (CAFs)-mediated ECM remodeling	Tumor-derived signals activate CAFs, which deposit ECM components and secrete matrix remodeling enzymes.	Creates fibrotic stromal barriers, elevates IFP and promotes immunosuppressive tumor microenvironment.	CAF-rich tumors exhibit impaired immune cell infiltration and poor therapeutic response.	(24–32)
ECM architecture and tertiary lymphoid structures (TLSs)	Collagen/fibronectin organization regulates stromal niches and immune-cell positioning.	Appropriate stromal organization supports TLSs maturation and local immune cell activation, whereas excessive fibrosis disrupts TLSs function.	Mature TLSs correlate with increased lymphocyte infiltration and improved immune checkpoint inhibitors (ICIs) response	(33–35, 186–188)
Cell stiffness	Cortical actin remodeling, RhoA/ROCK/myosin II regulation and cytoskeletal adaptation collectively regulate cellular deformability.	Tumor cell softening reduces susceptibility to CTL-mediated killing	Softer malignant cells show enhanced migration and impaired CTL-mediated killing.	(36–50)
Solid stress	Tumor cell proliferation and ECM accumulation generate spatially heterogeneous compressive and tensile stresses.	Compresses blood vessels, induces hypoxia, and limits immune cells infiltration and drug delivery	Elevated solid stress contributes to T cell exclusion and therapeutic resistance	(51–53)
Fluid shear stress	Abnormal tumor vasculature generates heterogeneous and disturbed blood flow conditions	Alters endothelial adhesion molecular expression, dendritic cells function and immune cell trafficking	Aberrant fluid shear stress reduces effective immune recruitment.	(54–62)
Interstitial fluid pressure (IFP)	Vascular leakiness, lymphatic dysfunction and ECM remodeling elevate IFP pressure.	Limits antibody penetration, immune cell migration and therapeutic delivery.	High IFP is associated with poor treatment response.	(10, 63–70)

### 2.1 ECM structure and stiffness

ECM stiffness is a biomechanical hallmark of solid tumors that contributes to tumor progression, metastasis, immune evasion, and therapeutic resistance. The ECM is primarily composed of 3 major classes of macromolecules: structural fibrous proteins, such as collagen and elastin, adhesive glycoproteins, such as fibronectin and laminin, and proteoglycans containing glycosaminoglycan, which exist either as free polysaccharides, such as hyaluronic acid, or as components of proteoglycans. In solid tumors, the ECM structure is extensively reprogrammed with aberrant composition and increased abundance of matrix macromolecules (13). Compared with those in normal tissues, collagen fibers in tumors often become thicker, denser and more highly aligned (14). Elastin fibers within the surrounding stroma contribute to tensile strength and elasticity of the ECM. In lung squamous cell carcinoma, elevated elastin deposition and its crosslinking with collagen help enhance ECM stiffness (15). In parallel, fibronectin forms fibrillar networks through integrin dependent assembly and provides a scaffold for the deposition of multiple ECM proteins, including collagen, fibrillin and latent TGF-β binding proteins (16). Laminins are key components of the basement membrane and connect to collagen IV to form a highly ordered structure within the basement membrane (17). In solid tumors, many of these components are upregulated or spatially redistributed, producing a matrix that is denser, more fibrotic and more permissive for tumor survival and immune cell migration restriction (18). Proteoglycans also contribute substantially to this process. For example, perlecan binds to ECM proteins to support basement membrane and regulates ECM related gene expression, thereby promoting ECM stiffness (19). Hyaluronic acid (HA) is a unique non-proteinaceous ECM component, which can interact with other components to form complex network large aggregates that contribute to ECM stiffness.

#### 2.1.1 ECM stiffness heterogeneity in normal and tumor tissues

The structural alterations in the ECM are a major driver of ECM stiffness. In normal tissue, ECM varies markedly among different tissues and spans several orders of magnitude. Soft tissues, such as the brain, adipose tissue, lung parenchyma and liver, generally exhibit elastic moduli ranging from several hundred pascals to several kilopascals, whereas skeletal muscle typically displays a stiffness of approximately 8–20 kPa (20). Similarly, ECM stiffness also varies markedly across cancer types. For example, triple-negative breast cancer is commonly characterized by collagen-rich, hyaluronan-enriched matrices with stiffness values exceeding 5–8 kPa (21). By contrast, the ECM of intrahepatic cholangiocarcinoma is almost twice as stiff as that of hepatocellular carcinoma (22). These changes are not merely structural, but also functionally important, as increased ECM stiffness can promote the formation of an immunosuppressive TME (23). However, these values should be regarded as representative ranges because apparent tissue stiffness is strongly influenced by anatomical location, loading conditions, measurement scale and experimental methodology.

#### 2.1.2 Desmoplastic reactions and cancer associated fibroblasts-mediated ECM remodeling

Desmoplastic reactions (DRs) refer to the formation and expansion of fibrous or connective tissue components within the stromal regions invaded by cancer cells. This reaction is characterized by fibroblast activation, excessive deposition of ECM components, and disorganized vasculature. It occurs across a wide range of malignancies, including breast cancer, biliary tract cancers and pancreatic ductal adenocarcinoma, and contributes to tumor progression, invasion, metastasis and resistance to anticancer therapies (24–26). In this process, cancer associated fibroblasts (CAFs) are the principal drivers of tumor ECM remodeling, serve as key cellular mediators of tumor ECM remodeling (27). They are activated by tumor derived factors, such as transforming growth factor-β (TGF-β) and platelet-derived growth factor (PDGF) and subsequently produce large amounts of matrix proteins (28, 29). At the same time, they also secrete matrix modifying enzymes such as lysyl oxidase (LOX) and lysyl oxidase‐like 2 (LOXL2), which further strengthen the matrix by increasing collagen crosslinking (30). LOX plays a particularly important role in the regulation of ECM stiffness (31). Studies indicate that LOX promotes collagen deposition and crosslinking in breast cancer, whereas inhibition of LOX reduces collagen crosslinking and lowers ECM stiffness (32). Collectively, these findings highlight that DRs not merely passive histological features of tumor stroma but also represent an active and dynamic process that reshapes the biomechanical landscape of the TME. Therefore, targeting DRs and their mechanical consequences, rather than simply reducing ECM abundance, may provide a more effective strategy to improve cancer therapy.

#### 2.1.3 ECM architecture organizes tertiary lymphoid structures and local antitumor immunity

Tertiary lymphoid structures (TLSs) are ectopic lymphoid aggregates that typically comprise distinct T-cell zones enriched in antigen presenting dendritic cells (DCs) and B cell follicles containing germinal centers (33). Through coordinated interactions among immune and stromal compartments, mature TLSs provide local sites for antigen presentation, T-cell priming, B-cell maturation, and antibody production, thereby supporting sustained antitumor immunity. Importantly, stromal cells together with collagen and fibronectin fibers provide a scaffold that regulates immune cell migration, antigen presentation and sustained cell interactions (34). Excessive collagen deposition and crosslinking generate dense and rigid matrices that reduce pore size, restrict lymphocyte trafficking, and contribute to immune evasion (35). Thus, ECM architecture actively shapes whether the tumor stroma functions as an immune excluding barrier or an immune supportive niche.

### 2.2 Cell stiffness

Unlike ECM, cell stiffness is determined by intracellular and cell surface associated factors, including nucleus mechanics, osmotic pressure and the cellular coat, and, in particular, the cortical actin cytoskeleton (36–38). The actin cortex, a thin, highly dynamic and cross-linked actomyosin network located immediately beneath the plasma membrane, is a key determinant of cell shape, deformability and resistance to external mechanical forces (39, 40). Resting immune cells, including T cells and neutrophils, are generally reported to exhibit elastic moduli in the sub-kilopascal range (approximately 0.1–1 kPa), whereas adherent stromal cells such as fibroblasts often display values of approximately 2–10 kPa (41).

Many malignant cells from multiple cancer types exhibit reduced stiffness compared with their normal counterparts, probably owing to remodeling of the cortical actin cytoskeleton rather than simply reflecting a generalized loss of cellular structural integrity (42–44). During malignant progression, cortical filamentous actin (F-actin) may become less uniformly organized, while stable cortical bundles and stress fibers are redistributed into highly dynamic actin structures, including lamellipodia, filopodia, invadopodia, and membrane blebs (45, 46). Dysregulation of actin-related protein 2/3 complex (Arp2/3), cofilin dependent filament severing, actin crosslinking, and RhoA/ROCK/myosin II contractility can further alter cortical thickness, cortical tension, and membrane cortex attachment (46). Collectively, these changes can reduce cell stiffness, thereby facilitating migration and metastasis. Consistent with this concept, metastatic cancer cells isolated from the body fluids of patients can exhibit substantially reduced stiffness than corresponding benign cells (47). In ovarian cancer, lower cell stiffness is associated with greater invasive and metastatic potential, whereas experimentally increasing cell stiffness suppresses invasion (48). Similarly, breast cancer stem cells are softer than their parental cells, with similar observations reported in colorectal cancer (49, 50). However, tumor cell softening should not be viewed solely as a consequence of malignant transformation. It may also represent an adaptive mechanical state that enables tumor cells to accommodate changes in the TME.

### 2.3 Solid stress

Solid stress refers to the mechanical stress borne by the solid phase of a tissue, including tumor cells, stromal cells, and ECM. Compressive and tensile stresses represent mechanically distinct components of solid stress. Compressive stress pushes tissue components together and produces local shortening or compaction, whereas tensile stress pulls them apart and causes tissue stretching. These two stress states also have different biological consequences. Compressive stress in the tumor interior can collapse blood and lymphatic vessels, thereby reducing perfusion, aggravating hypoxia, and limiting immune cell infiltration and drug delivery (51, 52). By contrast, tensile stress at the tumor periphery can stretch and align collagen fibers and induce ECM stiffening, generating migration tracks that may facilitate local invasion into the surrounding tissue (53). Nevertheless, the direct biological effects of tensile stress remain less extensively characterized than those of compression and require further experimental investigation.

### 2.4 Fluid shear stress

Fluid shear stress represents another key biophysical feature of solid tumors and plays a critical role in regulating tumor progression and metastasis (54). In normal tissues, fluid shear stress is relatively stable. In normal arteries, shear stress typically ranges from approximately 10 to 20 dyn/cm^2^, whereas smaller vessels and microcirculation generally experience lower but relatively constant shear forces of approximately 1 to 10 dyn/cm^2^ (55). In tumors, however, fluid shear stress is disrupted by tortuous, dilated and leaky vessels, incomplete endothelial structure, poor pericyte coverage, and vessel compression (56). As a result, the TME is characterized by highly heterogeneous shear conditions, with reported fluid shear stress values typically ranging from 0.1 to 35 dyn/cm^2^, generally lower and more irregular than those in normal vessels (57, 58). These vascular abnormalities also reduce and destabilize intratumoral flow, producing a spatially and temporally heterogeneous shear landscape, usually 0.001 to 10 mm/s, far below those in normal vessels (59). Fluid flow exerts shear stress on endothelial mechanosensors, a process that is essential for maintaining vascular health. Under physiological conditions, normal fluid shear stress helps maintain endothelial function and supports immune cell adhesion and migration (60). However, the abnormal architecture and high permeability of tumor neovessels further aggravate these disturbances, generating a microenvironment in which fluid flow and shear conditions are highly abnormal. Such changes are functionally important because fluid mechanical cues directly influence immune cell behavior. Studies have shown that low fluid shear stress impairs the motility and cytotoxic function of natural killer (NK) and CD8+T cells (61). In addition, DCs exhibit defective cytoskeletal reorganization and reduced antigen presenting capacity under these conditions, collectively weakening effective antitumor immune responses (62). Collectively, these findings suggest that vascular normalization in the TME may provide new opportunities to improve the efficacy of cancer immunotherapy.

### 2.5 Interstitial fluid pressure

IFP is an important determinant of tumor progression and therapeutic response. In normal tissues, IFP is usually maintained close to atmospheric pressure, typically within approximately -3 to +3 mmHg through a dynamic balance among capillary filtration, lymphatic drainage, and the osmotic and hydrostatic properties of the ECM (10). By contrast, in solid tumors, this balance is profoundly disturbed by abnormal blood vessels, defective or compressed lymphatic drainage, ECM remodeling, and progressively increasing solid stress, all of which contribute to elevated IFP (63). As a result, many solid tumors, including those of the head and neck, breast, and cervix, exhibit markedly increased IFP, typically ranging from 5 to 40 mmHg (64–67). The pressure difference between the tumor and surrounding normal tissue creates a steep gradient at the tumor margin, driving outward interstitial flow from the tumor core toward the periphery (68). This abnormal fluid movement not only facilitates tumor cell migration and invasion into adjacent tissues and lymphatic structures but also disrupts fluid dynamics within the TME. At the same time, elevated IFP acts as a physical barrier to treatment by reducing the capillary transport of therapeutic agents and limiting their penetration into tumor tissue (69). Notably, IFP does not act in isolation. Solid stress can further increase IFP by compressing blood and lymphatic vessels, whereas elevated IFP may in turn enhance solid stress through flow induced matrix reorganization, indicating a bidirectional relationship between these 2 mechanical abnormalities (70). Although recent studies have begun to examine IFP across different tumors, its overall landscape and dynamic interactions with other mechanical features of the TME remain insufficiently characterized and require further investigation.

### 2.6 Bidirectional crosstalk between hypoxia and abnormal mechanical properties

Mechanical abnormalities within the TME promote hypoxia when solid stress and ECM stiffening compress vessels and when elevated IFP reduces effective perfusion (51, 68). Hypoxia then activates hypoxia-inducible factor 1α (HIF-1α), which promotes collagen expression, angiogenesis, and ECM remodeling (71). In addition, HIF-1α increases TGF-β and LOX family activity to promote collagen crosslinking and recruit myeloid cells to premetastatic niches, while hypoxia tumor regions exhibit disrupted collagen organization and impaired macromolecular transport (72, 73). Therefore, mechanical abnormalities within the TME are not only an upstream driver of hypoxia, but also a downstream result of hypoxic remodeling. From this perspective, disrupting this reciprocal interaction between mechanical properties and hypoxia may represent a promising strategy to improve anticancer therapeutic responses.

## 3 Mechanical properties regulate cancer immunity cycle to induce immunotherapy resistance

**Figure 2:**
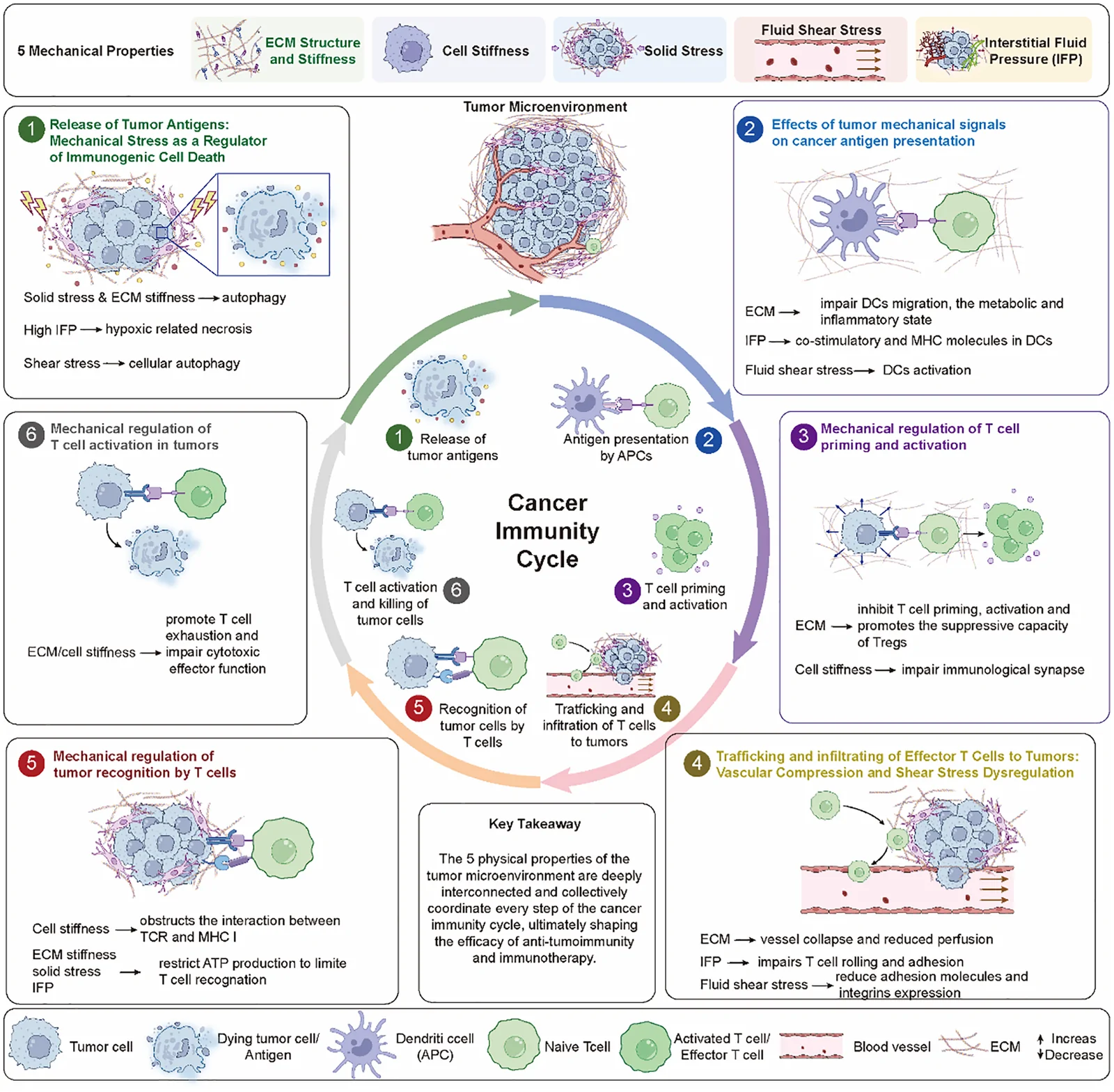
Mechanical abnormalities in the tumor microenvironment establish mechanical immune checkpoints and promote resistance to immunotherapy. The figure was created using Adobe Illustrator.

The efficacy of immunotherapy mainly depends on the generation of effective antitumor immune responses to eliminate cancer cells throughout the cancer immunity cycle, together with coordinated systemic responses that ensure the efficient delivery of therapeutic antibodies to the TME (74). However, alterations in the mechanical properties of the TME can disrupt the cancer immunity cycle, thereby contributing to immunotherapy resistance (**Figure 2**).

### 3.1 Release of tumor antigens: mechanical properties as regulators of immunogenic cell death

The release of tumor associated antigens is the first step of the cancer immunity cycle. During immunogenic cell death (ICD), tumor associated antigens are released together with damage associated molecular patterns (DAMPs), including extracellular ATP and high mobility group box 1 protein (HMGB1), whereas calreticulin is exposed on the surface of dying tumor cells. These signals recruit and activate antigen-presenting cells and present on major histocompatibility complex class I (MHC-I) molecules to CD8^+^ T cells (75). Importantly, the ICD cannot be inferred solely from its morphological classification. Apoptosis can be either immunologically silent or immunogenic, whereas autophagy and necrosis can exert both immunostimulatory and immunosuppressive effects (76–78).

Autophagy is primarily an intracellular recycling process rather than a distinct mode of cell death. In pancreatic cancer, autophagy can impair immune surveillance by promoting lysosomal degradation of MHC-I molecules, thereby reducing tumor-antigen presentation and recognition by cytotoxic T lymphocytes (79). In this context, autophagy may therefore promote tumor cell survival, immune evasion and immunotherapy resistance.

Necrosis is similarly context-dependent. Acute necrosis causes plasma membrane rupture and the release of tumor antigens and DAMPs. For example, HMGB1 can activate TLR4 signaling in DCs and promote the processing and presentation of tumor associated antigens, whereas extracellular ATP can activate NLRP3 signaling and IL-1β production, facilitating CD8^+^ T cell priming (80, 81). However, extensive or persistent necrosis within poorly perfused tumor regions may instead generate chronic inflammation. Prolonged release of HMGB1, IL-1α, nucleic acids, and other inflammatory mediators can recruit tumor-associated macrophages (TAMs), neutrophils, and myeloid derived suppressor cells, while promoting angiogenesis, tissue remodeling, and immune suppression (82). Under these conditions, necrosis may support tumor progression rather than productive immune surveillance.

Mechanical abnormalities within the TME may influence this process by inducing adaptive autophagy or promoting necrosis. For example, exposure of HeLa cells to 0.773 kPa of solid stress induced autophagy and enhanced invasion by facilitating paxillin turnover and metalloproteinase 2 (MMP-2) secretion (83). Mechanical compression also rapidly induced autophagosome formation in *Dictyostelium* cells exposed to approximately 1.15 kPa and in MDA-MB-231 breast cancer cells exposed to 0.25 kPa, indicating that autophagy represents a conserved adaptive response to mechanical perturbation (84). Similarly, fluid shear stress induced protective autophagy in HeLa cells (85), whereas promoted autophagy and migration in hepatocellular carcinoma cells (86). In T24 bladder cancer cells, a flow protocol of 0.03 mL/min for 3 h altered the ER stress/cytoskeleton axis and autophagic competence (87). Notably, 0.03 mL/min represents the experimental flow rate rather than the actual shear stress. In parallel, enhanced solid stress and ECM expansion can compress tumor vessels, leading to hypoperfusion, hypoxia, and nutrient deprivation and ultimately promoting necrosis in poorly vascularized tumor regions (51). However, these studies did not directly assess DCs activation or T-cell priming in the TME.

### 3.2 Effects of tumor mechanical signals on tumor antigen presentation

DCs are highly efficient antigen presenting cells that link innate and adaptive immunity. They capture tumor associated antigens and present them to T cells through MHC molecules (88). In addition to cancer antigens, DCs can also be activated by inflammatory cytokines, including IL-1β, TNF-α, which promote their differentiation and maturation (89). By contrast, inhibitory cytokines within the TME, including IL-10 and TGF-β, suppress these processes, leading to “cold” tumors that lack effective immune priming (90). Increasing evidence suggests that mechanical signals within the TME influence DC function. Elevated IFP has been reported to increase the expression of co-stimulatory and MHC molecules and IL-12 in DCs (91), although the implications of these changes for tumor immunosurveillance remain unclear. ECM structure is another important regulator. Studies have shown that collagen crosslinking and the ECM remodeling impair the differentiation, antigen uptake capacity and migration of immature DCs (iDCs) (92). However, another study showed that hydrogel-based tumor ECM had minimal effects on mature DCs, which maintained uniform differentiation and functional profiles across even under high ECM stiffness. These findings suggest that the effects of ECM structure and stiffness on DCs function may depend on the maturation stage.

In addition, ECM stiffness can directly alter the metabolic and inflammatory state of DCs. DCs cultured on stiff hydrogel displayed increased activation of major glucose metabolic pathways, together with elevated secretion of IL-1α, IL-1β, IL-6, IL-12, and tumor necrosis factor-α (TNF-α) (93). Fluid shear stress also appears to be involved in DCs regulation. Exposed to low levels of fluid shear stress (0.2-0.6 dyn/cm^2^) increased the expression of DC activation markers, including MHC-I and CD86 (94). However, the molecular mechanisms underlying how DCs respond to mechanical properties remain incompletely understood.

### 3.3 Mechanical regulation of T cell priming and activation

Following cancer antigen capture, effector T cells are primed and activated against the tumor specific antigens. During this process, T cells are exposed to dynamic mechanical environment, and increasing evidence indicates that mechanical properties directly or indirectly influence T cell priming and activation (95–97). For example, T cell proliferation was significantly reduced when cells cultured in 3-dimensional conditions with high collagen density (98). Consistently, softer (Young’s Modulus < 100 kPa) substrates promote *ex vivo* T cell proliferation and IL-2 production based upon solid-phase immobilization of TCR/CD3 and CD28 ligands (99). In addition, excessive deposition of hyaluronan and collagen has been reported to inhibit T cell priming in tumor draining lymph nodes (100).

Mechanical regulation also extends to the formation of the immunological synapse (IS), which forms when cytotoxic T lymphocytes scan antigen presenting cells or target tumor cells (101). Recent work by Renping Zhao and colleagues showed that reduced cell stiffness impaired immunological synapse formation and inhibited NFAT1 nuclear translocation in T cells (102), suggesting that target-cell mechanics can directly influence T cell activation signals. Beyond effects on effector T cells, mechanical cues also appear to strengthen suppressive immune populations.

Mechanically polarized regulatory T cells (Tregs) can inhibit T cell priming by preventing IS maturation in naïve T cells (103). Tregs also exhibit distinct mechanosensitivity characterized by elevated expression of YAP (104). In a stiff ECM environment, YAP-dependent transcription enhances FOXP3 stability and increases expression of the inhibitory receptor PD-1 (105), indicating that ECM stiffness may simultaneously restrain effector T cell activation and enhance the immunosuppressive function of Tregs.

### 3.4 Trafficking and infiltration of effector T cells to tumors: vascular compression and shear stress dysregulation

Activated T cells subsequently traffic to and infiltrate the TME to kill tumor cells. The efficacy of cancer immunotherapy largely depends on this step (106). During this process, activated T cells undergo a cascade of selectin-, integrin- and chemokine-dependent interactions that coordinate rolling, firm adhesion, transmigration and interstitial motility. Although chemokine gradients, adhesion molecules and vascular cues define the canonical paradigm of T cell recruitment (107), studies over the past few years have revealed that biophysical and mechanical cues within the TME exert equally important control over T cell trafficking (108).

Endothelial cells within the TME serve as key gatekeepers of immune cell infiltration. In normal vessels, fluid shear stress (1 to 50 dyn/cm (2)) generated by blood flow maintains endothelial quiescence through KLF2 dependent transcription (109–112), promoting the expression of adhesion molecules and integrins, including ICAM-1, VCAM-1, VLA-4 and LFA-1 that support T-cell adhesion (113, 114). In tumors, however, vessels are exposed to low magnitude, and oscillatory shear stress, leading to endothelial dysfunction, loss of polarity, and abnormal fluid shear stress (115, 116). In this context, disturbed fluid shear stress downregulates the expression of ICAM-1 and E-selectin, 2 key adhesion molecules involved in T cell recruitment, in endothelial cells in a time dependent manner (117). In addition, elevated ECM stiffness compresses capillaries, thereby reducing perfusion and oxygen delivery, activating hypoxia-inducible factor 1α (HIF-1α) and promoting VEGF release (68). VEGF not only increases vascular leakiness but also suppresses endothelial adhesion molecules via autocrine signaling, further impairing T cell infiltration (118, 119). In gastrointestinal cancer mice models, mechanical decompression via targeted hyaluronan degradation reshaped ECM, leading to improved infiltration of CD8^+^ T cells (120). Studies also showed that collagen density in the TME suppresses immune cell infiltration, as reflected by the paucity of T cells within breast cancer tissues with high collagen density (98). In parallel, abnormal ECM structure around vessels increases barrier resistance, further restricting transmigration of CD8+T cells (52, 121). However, direct evidence that alterations in mechanical signals within the TME contribute to immunotherapy resistance remains limited.

### 3.5 Mechanical regulation of CD8+ T cell‑mediated tumor recognition

Recognition of tumor cells by CD8+ T cells is a central step in antitumor immunity. Accumulating evidence indicates that mechanical properties play critical roles in tumor immune recognition by shaping T-cell engagement with tumor cells and determining the efficiency of cytotoxic responses (122, 123). A key event in tumor recognition is the interaction between the TCR and MHC-I. Disruption of TCR/MHC-I signaling within the TME can impair antitumor immunity and facilitate cancer cell immune evasion (124). Notably, mechanical remodeling of the TME can directly affect this axis. Increased ECM stiffness in prostate cancer reduces MHC-I expression through the USP8/NBR1 axis, thereby weakening tumor cell recognition and contributing to immunotherapy resistance (125). Mechanical regulation of tumor recognition also occurs at the level of membrane organization. The glycocalyx, which is often highly expressed on the surface of cancer cells, can regulate membrane morphology and induce cytoskeletal rearrangement, thereby decreasing cell stiffness (126, 127). This structure forms a physical barrier that hinders TCR engagement. Consistent with this, studies have shown that glycocalyx on the cell membrane effectively obstructs the interaction between TCR and MHC-I (128). In pancreatic cancer, targeting the glycocalyx has been reported to enhance CAR-T activation in a density- and affinity-dependent manner (129). In parallel, cellular metabolism caused by abnormal mechanical properties may further compromise tumor recognition. In addition, hypoxia, nutrient deprivation, and acidosis caused by increased ECM stiffness, solid stress and IFP restrict ATP production, thereby limiting the recognition ability of T cells (130–132).

### 3.6 Mechanical regulation of CD8+ T cell activation in tumors

Once recognition occurs, cytotoxic T lymphocytes (CTLs) undergo polarization, secrete cytokines and overcome target cells’ defense mechanisms to eliminate tumor cells. It is well established that mechanical properties within the TME regulate T cell function, including T cell exhaustion, the pore-forming activity of perforin, and immune synapse formation and stability (133).

T cell exhaustion, a hallmark of ineffective antitumor immunity, is characterized by reduced effector function, impaired cytotoxic secretion and sustained expression of inhibitory receptors (134). Mechanical abnormalities within the TME can shift signaling towards inhibitory immune states. For example, increased ECM stiffness has been reported to promote T cell exhaustion through a coupled mechanism involving TCR signaling and Piezo1/calcium/CREB axis (135). In non-small cell lung cancer, matrix stiffening also activates Piezo1 in tumor cells, which promote expression of PD-L1 (136). These findings may help to explain why checkpoint blockade often shows limited efficacy in mechanically hostile tumors (137).

Effective tumor cell killing requires the formation of a stable immunological synapse, a mechanically active interface that coordinates receptor signaling, adhesion, and release of cytotoxic granules by CTLs or NK cells (138). At this interface, WASP/Arp2/3-dependent actin protrusions exert forces on and deform the target-cell surface while coordinating perforin secretion (139). Perforin forms pores in the target cell membrane, allowing granzymes to enter the cell and induce apoptosis (140, 141). Importantly, the effects of matrix stiffness on cytotoxic lymphocyte function are context-dependent rather than uniformly stimulatory or inhibitory. In 2-dimensional systems, increasing substrate stiffness within certain ranges can enhance TCR signaling and IL-2 production (142). However, softer substrates have also been reported to support greater CD8+ T cell proliferation than extremely rigid substrates, indicating that the response depends on the stiffness range, material composition, ligand density, and activation state of the lymphocyte (99). Collagen deposition and crosslinking generally increase matrix stiffness while simultaneously reducing pore size. High density collagen suppresses CTL proliferation and cytotoxic phenotype and markedly reduces CTL migration even when the expression of perforin and granzymes remains intact (4). Thus, an excessively stiff and poorly porous matrix primarily limits cytotoxicity by restricting lymphocyte infiltration and target-cell access rather than by directly preventing the molecular action of perforin.

Once physical contact with a tumor cell is established, mechanical properties of the target cell become more directly relevant to cytotoxicity. In particular, cortical stiffness, membrane tension, and actomyosin support can influence force transmission at the immunological synapse and the efficiency of perforin pore formation. Consistent with this concept, MRTF-induced rigidification of the F-actin cytoskeleton increased the susceptibility of melanoma and breast cancer cells to killing by both CTLs and NK cells (143). Conversely, mechanically soft tumor cells were more resistant to CTL killing because reduced F-actin support impaired perforin pore formation and granzyme B entry (42). Cholesterol depletion also stiffened the tumor cells, increased forces at the immunological synapse, and enhanced adoptive T-cell therapy in mouse tumor models (144). Beyond perforin activity, softer tumor cells impair MTOC reorientation and NFAT1 nuclear translocation through distinct Ca^2+^ dependent pathways involving PIEZO1 (102). Moreover, hyperglycemia-induced cholesterol accumulation can soften the tumor cell plasma membrane and reduce CD8^+^ T cell cytotoxicity (145). Collectively, these findings suggest that ECM primarily regulates immune-cell access and migration, whereas target cell stiffness more directly influence synapse function and perforin activity.

Mechanical regulation of tumor immunity also extends beyond direct tumor-lymphocyte interactions. TAMs serve as pivotal intermediaries that translate mechanical signals into biochemical immunosuppression (146). Elevated ECM stiffness can bias macrophage polarization towards TAM2 through integrin αvβ3/FAK/YAP signaling, thereby enhancing the production of TGF-β and IL-10 (147). These cytokines suppress T cell priming by impairing antigen presentation by DCs (148). Stiff matrices can also activate macrophage mechanosensors, including TRPV4 and Piezo1, leading to Ca^2+^ influx and increased arginase-1 expression, which further limits T cells proliferation (97). By contrast, within a softer ECM, macrophages tend to polarize TAM1 that supports T cell activation through the production of IL-12 and TNF-α (149). Elevated solid stress can further suppress the cytotoxic activity of NK cells while inducing immunosuppressive signals such as TGF-β, which in turn promotes the expansion of regulatory T cells (Tregs) and TAM2 (150–152).

Neutrophils also contribute to the mechanically regulated immune landscape. Under conditions of elevated fluid shear stress and ECM stiffness, neutrophils release neutrophil extracellular traps (NETs), which consist primarily of extracellular DNA, histones, and associated proteins (153, 154). These structures may form physical barriers that restrict T-cell motility and impair antigen priming. NETs associated components, including fibronectin fragments and citrullinated histones, may also activate integrin signaling in T cells and promote dysfunction states of T cells. *In vitro*, NET-decorated stiff matrices reduce CD69 and IL-2 expression by over 50% compared to compliant environments, demonstrating the suppressive role of neutrophil-derived mechanical scaffolds (155).

## 4 Therapeutic strategies targeting the mechanical properties of the TME: current evidence and limitations

The mechanoimmunology field has broadened our understanding of tumor immunotherapy, highlighting that immune failure in solid tumors is often caused not only by insufficient immune activation, but also by mechanical properties within the TME. As a result, therapeutic strategies targeting mechanical properties have emerged as a promising and complementary way to improve the response to immunotherapy (**Table 2**).

**Table 2:** Therapeutic strategies targeting the mechanical properties of the TME to enhance immunotherapy efficacy discussed in this review.

Therapeutic strategy	Molecular/mechanical targets	Mechanistic rationale	Current evidence and limitations	References
ECM softening and collagen crosslinking inhibition	LOX/LOXL2 inhibition, collagen deposition and crosslinking; β-aminopropionitrile and the anti-LOXL2 antibody simtuzumab.	Reduces ECM stiffness and improves immune cell penetration.	LOX/LOXL2 inhibition improves immune infiltration and treatment response in preclinical models. However, simtuzumab failed to improve clinical outcomes in randomized trials, highlighting pathway redundancy, context dependence and the need for biomarker guided patient selection.	(156–165)
CAF-targeted therapy	TGF-β, FAK, Hedgehog, FAP pathways	Limits collagen, fibronectin and hyaluronan deposition; reduces matrix contraction and TGF-β/CXCL12-mediated immunosuppression; decompresses vessels; and improves CD8^+^ T-cell access.	Evidence remains predominantly preclinical. CAF heterogeneity limits non-selective depletion strategies	(166–185)
TLS-preserving stromal modulation	Podoplanin-positive lymphoid tissue organizer-like fibroblasts, reticular stromal networks, CXCL13/CCL21 chemokines and lymphotoxin–LTβR signaling.	Maintains immune-supportive niches while reducing pathological fibrosis.	Mature TLSs are consistently associated with favorable outcomes and improved responses to ICIs, but therapeutic TLSs induction remains largely preclinical. TLS maturity, location and cellular composition require spatially resolved assessment.	(186–188)
Vascular normalization and IFP reduction	VEGF/VEGFR signaling, bevacizumab, antiangiogenic strategies	Improves perfusion, decreases IFP and enhances immune-cell trafficking	Losartan-containing regimens have produced encouraging phase II results, although the specific contribution of mechanical normalization remains uncertain. PEGPH20 failed to improve survival in phase III pancreatic cancer. Antiangiogenic therapy requires an appropriate normalization window because excessive vessel pruning can aggravate hypoxia and immune exclusion.	(189–196)
Mechanotransduction pathway inhibition	Integrin/FAK, RhoA/ROCK/myosin II, YAP/TAZ	Suppresses stiffness-induced CAF activation, production, tumor cell invasion and PD-L1 expression, while potentially restoring immune-cell trafficking and cytotoxic function.	Most evidence is preclinical. These pathways are pleiotropic and partly redundant, and their inhibition can produce opposing effects in tumor, stromal and immune cells. Systemic toxicity and cell-type specificity remain major translational challenges.	(197–204)
Mechanosensitive ion channel targeting	Piezo1/Ca^2+^ signaling	Reprograms mechanical sensing in tumor, endothelial and immune cells, with potential effects on T-cell activation or exhaustion, macrophage polarization, vascular function and tumor-cell PD-L1 expression.	Evidence is at an early preclinical stage. Channel functions vary by cell type, mechanical stimulus and disease stage, whereas systemic blockade may disrupt physiological cardiovascular and neural mechanosensing. Targeted delivery will probably be required.	(205–208)
Mechano-immunoengineering approaches	Mechanically tunable three-dimensional hydrogels, decellularized matrices, patient-derived organoids, tumor-on-chip and microfluidic systems, and mechanically responsive biomaterials or nanocarriers.	Enables controlled modelling of stiffness, viscoelasticity, stress relaxation, shear stress and pressure; reconstructs tumor-stromal-immune interactions; supports patient-specific treatment testing; and may permit spatially controlled therapeutic delivery.	Current applications are mainly proof of concept or preclinical. Key barriers include poorly defined matrix materials, batch variability, incomplete immune and vascular compartments, insufficient standardization of mechanical endpoints, manufacturing constraints and limited prospective clinical validation.	(209–211)

### 4.1 LOX-mediated ECM remodeling and its therapeutic potential in cancer immunotherapy

Increasing evidence suggests that reducing collagen crosslinking can lower ECM stiffness, facilitating CD8+ T cell migration and infiltration (156). In line with this view, Vasyl Chekhun and colleagues reported that high PD-L1 expression in breast cancer tissue is associated with LOX, suggesting a link between matrix remodeling and tumor immune status (157). Further preclinical studies have shown that combined targeting of LOX and ICIs can reshape the immune microenvironment by increasing the infiltration of neutrophils, B cells and CD8+ T cells, providing a possible strategy to remodel melanoma and other tumor types and improve the efficacy of immunotherapy (158). Among LOX family members, LOXL2 has attracted particular attention due to its role in maintaining the abnormal stromal microenvironment in cancer (159). In preclinical studies, simtuzumab, an antibody precursor targeting LOXL2, has been shown to attenuate angiogenesis, suppress tumor growth and inhibit metastases (160–162). A phase I/IIa study reported tumor size reduction in patients with advanced solid tumors treated with simtuzumab (163). However, this promise was not confirmed in later trials. Two multicenter, randomized, double-blind, placebo-controlled phase II studies failed to show additional clinical benefit when simtuzumab was combined with chemotherapy in metastatic KRAS-mutant colorectal cancer or pancreatic ductal adenocarcinoma (164, 165). These findings suggest that targeting the LOX family can effectively normalize ECM, but translation of this strategy into clinical benefit remains challenging.

### 4.2 Targeting CAFs to enhance cancer immunotherapy

CAFs promote immunotherapy resistance mainly through ECM remodeling, growth factor secretion, and immune modulation, all of which contribute to elevated IFP (166–168). Recent studies have further identified CAFs as important promoters of an immunosuppressive TME, limiting the effectiveness of current immunotherapeutic strategies (169). As a result, targeting CAFs recruitment, activation, and related signaling pathways has emerged as a promising approach to improve immunotherapy responses (170, 171).

In solid tumors, transforming growth factor-β (TGF-β) is a driver of CAFs formation and maintenance (172–174). A recent preclinical study showed that the TGF-β receptor I inhibitor SB525334 markedly suppressed CAFs proliferation in pancreatic cancer mouse model, increased p53 protein expression, reduced collagen fiber deposition, and promoted microvessel normalization (175). Activation of TGF-β signaling in fibroblasts is also associated with poor immune cell infiltration and immune evasion (176). In mouse mammary carcinoma models, combining TGF-β blockade with PD-L1 inhibition improved antitumor responses by relieving T-cell exclusion (35). Several clinical trials have therefore investigated TGF-β targeted therapies in cancer. Among these, Vactosertib (TEW-7197), a highly selective and potent inhibitor of TGF-β receptor type 1, combined with pembrolizumab showed anti-tumor activity, prolonged overall survival and manageable safety profiles in patients with MSS mCRC in a phase 1b/2a study (NCT03724851) (177). Other agents, including NIS793, vactosertib (TEW-7197), and galunisertib (LY2157299), are also being evaluated for their potential to improve immunotherapy by reducing TGF-β mediated CAFs activation.

In addition to TGF-β inhibition, targeting focal adhesion kinase (FAK) or the Hedgehog pathway represents another strategy to restrain CAFs activity. Persistent FAK activation has been shown to maintain CAFs features and support ECM remodeling (178–180). Preclinical studies have demonstrated that FAK inhibition delays tumor growth, improved survival, and rendered tumors more responsive to immunotherapy in pancreatic ductal adenocarcinoma (PDAC) (181). Clinical observations also support stromal targeting, as lower fibroblast activation protein (FAP) expression in fibroblasts has been associated with greater CD8+ T cell and TAM1 infiltration in PDAC (182). A randomized phase II trial suggested that defactinib, an oral FAK inhibitor, combined with pembrolizumab as neoadjuvant therapy after chemotherapy, showed promising activity in patients with resectable PDAC (183).

The sonic Hedgehog signaling pathway has also been implicated in CAFs biology. Studies have shown that Hedgehog pathway is highly activated and contributes to PDAC growth and the establishment of an immunosuppressive TME (184). In preclinical models, inhibition of the Hedgehog pathway inhibition reduced proliferating CAFs and collagen and hyaluronan levels, significantly improving the efficacy of chemotherapy and nanotherapy (185). Several Hedgehog inhibitors, including vismodegib (GDC-0449), saridegib (IPI-926), and sonidegib, are currently being tested in clinical trials.

Importantly, therapeutic strategies targeting CAFs may also alter the formation and maturation of TLSs (186). Across melanoma, renal cell carcinoma, sarcoma and other malignancies, mature TLSs have been associated with enhanced local immune activation, increased lymphocyte infiltration, favorable clinical outcomes and improved responses to ICIs (33, 187, 188). However, indiscriminate depletion of CAFs or disruption of stromal networks may eliminate lymphoid tissue organizer-like fibroblasts and thereby impair TLSs maturation (186). Accordingly, future studies of CAFs-targeted therapies should assess TLS maturation, spatial organization and cellular composition alongside changes in extracellular matrix stiffness, tissue perfusion and immune-cell infiltration.

### 4.3 Targeting IFP and fluid shear stress to enhance antitumor immunity

Tumor blood vessels are often characterized by irregular fluid shear stress, vessel compression, and leakiness which elevate IFP and promote hypoxia in the TME (189). Anti-angiogenic and vascular normalization therapies can partially normalize vessel mechanics, leading to improved fluid shear stress, lower IFP, and reduced hypoxia (190). Among the pathways involved, VEGF/VEGFR signaling is one of the most important pathways that regulates angiogenesis and lymphangiogenesis (191). Bevacizumab, a VEGFA inhibitor, has been shown to reduce vascular permeability, improve vascular structure, and decrease IFP (192, 193). Clinical studies have further shown that combining bevacizumab with PD-L1 blockade can produce beneficial and synergistic effects in phase I to phase III trials (194). During vascular normalization, immune cells show improved adhesion, trafficking, and persistence, which may support more effective immune synapse formation and cytotoxic function (195, 196). However, an important challenge is to define and exploit this normalization window appropriately because excessive or prolonged vascular inhibition may worsen hypoxia, increase ECM stiffness, and ultimately strengthen immune suppression rather than relieve it.

### 4.4 Mechanotransduction pathways as therapeutic targets to enhance antitumor immunity

Beyond tissue-scale mechanics, tumor immunotherapy increasingly targets cell intrinsic mechanotransduction pathways that translate mechanical properties into immune dysfunction. Signaling pathways such as integrin/FAK, RhoA/ROCK/myosin II, and YAP/TAZ are now recognized as key regulators of immune synapse stability, cytoskeletal organization, or susceptibility to T cell exhaustion (197–200). In CD8+ T cells, high mechanical stress can activate these pathways in a way that increases baseline contractility and disrupts the local actin remodeling needed for granule polarization and secretion (201, 202). In tumor and stromal cells, these same pathways promote ECM deposition, PD-L1 expression, and immunosuppressive cytokine production (203, 204). However, because these pathways are widely expressed and have multiple functions in normal tissues, a major challenge is to modulate them in a way that enhances antitumor immunity without causing harmful systemic effects.

Mechanosensitive ion channels, particularly calcium channels have emerged as another important target at the interface between mechanical signaling and immune regulation (205). Chronic mechanical stress can disturb calcium signaling in CD8+ T cell (206). In experimental models, pharmacological targeting of these channels has shown potential to restore cytoskeletal balance and enhance T cell activity (207, 208). However, this approach remains at an early stage with challenges including insufficient channel specificity and the risk of interfering with basic mechanosensory functions in non-immune cells.

Given current evidence, the most promising application of mechanical properties in tumor immunotherapy likely lies in combination strategies. In this approach, mechanical normalization aims to reduce the physical and energy barriers that limit the efficacy of established immune therapies. By decreasing ECM stiffness, relieving solid stress, or normalizing vascular structure, biophysical interventions may restore immune synapse integrity, improve perforin and granzyme release, and delay the development of CD8+ T cell exhaustion, thereby enhancing responses to ICIs, adoptive cell therapy, or cancer vaccines. Importantly, this approach does not aim to replace immunotherapy. Instead, it is meant to support immunotherapy by creating conditions that help activated immune cells kill tumor cells more effectively and for a longer time.

## 5 Conclusion and perspective

In summary, targeting mechanical properties provides an important new framework for improving cancer treatment, suggesting that immunotherapy resistance should be understood not only at the molecular level, but also at a physical level. However, the mechanical regulation of antitumor immunity is highly dynamic and highly heterogeneous, whereas many current therapeutic strategies still focus only on single physical factor in isolation. In this context, patient-derived tumor organoids represent promising new approach methodologies. Nevertheless, it should not be regarded as mechanically faithful replicas of the native TME. Most organoids are cultured in basement membrane extract, which do not permit independent control of stiffness, viscoelasticity, stress relaxation, degradability, or matrix architecture. Mechanically tunable hydrogels can partly overcome these limitations by enabling matrix stiffness, viscoelasticity, degradability, and biochemical composition to be tailored to specific tumor tissues. Future organoid-based approach methodologies should therefore integrate tunable biomaterials, stromal and immune-cell co-cultures, and microfluidic perfusion, while being quantitatively benchmarked against matched patient tissues in terms of matrix composition, mechanical properties, spatial architecture, and treatment response.
