# Correction: Mechanical properties of the tumor microenvironment: drivers of immunotherapy resistance in solid tumors

**DOI:** 10.3389/fimmu.2026.1982199

**Published:** 2026-09-14

**Authors:** Zhen Zhang, Tianhao Deng, Wanshuang Zhou, Xiaoning Tan

**Affiliations:** Department of Oncology, The Affiliated Hospital of Hunan Academy of Chinese Medicine, Changsha, China

**Keywords:** cancer-immunity cycle, immunotherapy resistance, mechanical properties, mechanotransduction, tumor microenvironment

The funder the “This work was supported by the National Natural Science Foundation of China (Grant Nos. 82575174 and 82505707), the China Postdoctoral Science Foundation (Grant No. 2024M750887), the Hunan Provincial Health Science Research Project (Grant No. 20256746), the Chronic Disease Management Research Project of the Capacity Building and Continuing Education Center of the National Health Commission (Grant No. GWJJMB202510022217), the Changsha Outstanding Young Science and Technology Talent Training Program (Grant No. kq2506025), the Hunan Province Furong Health Talent Project (Xiang Wei Han [2024] No. 182), the Hunan Province 14th Five-Year Plan Backbone Talent Program (Xiang Zhong Yi Yao [2024] No. 3), and the 2025 Hunan Provincial Medical Discipline Category C Construction Project (Traditional Chinese Medicine Oncology) (Xiang Wei Yi Fa [2025] No. 7).” to all authors was erroneously omitted.

The original version of this article has been updated.

